# The intersection of liver cirrhosis and pulmonary fibrosis

**DOI:** 10.1186/s12967-025-07449-4

**Published:** 2025-12-26

**Authors:** Esref Alperen Bayraktar, Mary Salvatore

**Affiliations:** 1 Department of Radiology, Mayo Clinic, 200 1st St SW, Rochester, MN 55905 USA; 2 Department of Radiology, Jacobi Medical Center, NY Bronx, USA

**Keywords:** Cirrhosis, Idiopathic pulmonary fibrosis, Fibrosis, Cancer, Clinical trials

## Abstract

**Background:**

Liver cirrhosis and pulmonary fibrosis are fibrotic disorders that arise from chronic wound-healing processes in response to persistent injury. Despite involving different organs, both conditions share pathophysiologic mechanisms, such as fibroblast-to-myofibroblast transformation, extracellular matrix deposition, and impaired remodeling. These overlapping pathways suggest a common fibrogenic network that extends beyond the organs.

**Main Text:**

In cirrhosis, hepatocyte necrosis and chronic inflammation activate hepatic stellate cells that later differentiate into myofibroblasts and produce an altered extracellular matrix. Initially, matrix metalloproteinases counterbalance fibrosis. However, their activity is progressively inhibited by tissue inhibitors of metalloproteinases leading to excessive scarring, and regenerative nodules. Similarly, persistent alveolar epithelial injury disrupts regenerative capacity, causing maladaptive repair in pulmonary fibrosis. Fibrogenic mediators, particularly transforming growth factor-β and platelet-derived growth factor, drive fibroblast-to-myofibroblast differentiation and epithelial-mesenchymal transition. The resulting dense extracellular matrix creates a positive feedback loop that perpetuates fibrosis, leading to microscopic honeycombing and the histopathological pattern of usual interstitial pneumonia. Genetic and environmental factors cause the development of both conditions. Telomerase mutations and shortened telomeres predispose to cirrhosis and idiopathic pulmonary fibrosis. Smoking, hepatitis B and C are environmental factors for cirrhosis. On the other hand, epstein-barr virus, cytomegalovirus, and hepatitis C virus are responsible for pulmonary fibrosis. Both diseases promote vasoconstriction, leading to portal hypertension in the liver and pulmonary hypertension in the lungs. Transforming growth factor-β plays a dual role in carcinogenesis, acting as a tumor suppressor in early stages but later acquires tumor-promoting properties in both diseases.

**Conclusion:**

Cirrhosis and pulmonary fibrosis share molecular and cellular processes leading to fibrosis, vascular remodeling and malignant transformation in both diseases. Recognizing these overlapping mechanisms may help us better understand disease processes and guide development of targeted treatments.

## Background

Chronic damage to an organ, whether stemming from a disease or exposure to environmental factors, leads to cellular and molecular responses that cause fibrosis. Two of the most common types of organ fibrosis, liver cirrhosis, and pulmonary fibrosis, are associated with poor prognosis and high mortality. In this article, we compare these two diseases and highlight potential cross-disciplinary insights that experts in these fields can learn from one another.

Liver cirrhosis is the end result of many chronic liver diseases due to chronic wound healing [1]. Some pathologic features are common to all causes of liver cirrhosis, including deposition of extracellular matrix (ECM), necrosis of hepatocytes, replacement of liver parenchyma with regenerative nodules, and loss of liver function [2, 3]. Fibroblasts transform into myofibroblasts, which is crucial for fibrosis. Myofibroblasts produce an altered ECM [1, 4, 5]. Despite an initial competent ECM with fibrinolysis using matrix metalloproteinases, fibrogenesis eventually overcomes fibrinolysis by upregulating tissue inhibitors of matrix metalloproteinase (TIMP) inhibitors [4]. Resulting scar tissue, containing a complex arrangement of ECM molecules, basement membrane collagen type IV, non-collagenous glycoproteins, elastic fibers, glycosaminoglycans, proteoglycans, etc., replaces functioning hepatocytes [6]. Ultimately, these processes result in advanced scarring, microscopic cyst formation, and the histopathological pattern of cirrhosis.

Pulmonary fibrosis is a chronic, progressive disease resulting from abnormal remodeling in response to chronic wound healing [7, 8]. Genetic mutations, in combination with environmental exposures, lead to epithelial damage and dysfunction [8, 9]. The persistent injury overwhelms the ability of type 2 alveolar epithelial cells (AEC) to regenerate, resulting in maladaptive repair mechanisms [9]. Subsequently, fibrotic chemokines are released, causing fibroblast-myofibroblast differentiation and epithelial cell transformation into mesenchymal cells, which produce collagen and other ECM components [10, 11]. These dysregulations disrupt the organization of the ECM, resulting in a denser matrix [8, 9]. While balance and negative feedback loops are essential for successful wound healing, the stiffer matrix creates a positive feedback loop that increases myofibroblast activity and enhances fibrosis [8, 9, 12]. Ultimately, these processes result in advanced scarring, microscopic honeycombing, and histopathological patterns of usual interstitial pneumonia (UIP) [8, 9, 13].

## Hepatic lobule vs secondary pulmonary lobule

### Normal anatomy of the hepatic lobule and effect of cirrhosis

The hepatic lobule is the functional unit of the liver. This hexagonal structure is surrounded at the corners by portal triads (consisting of the hepatic artery, portal vein, and bile duct), and in the center is the hepatic vein. Inside, the chords of hepatocytes are divided into three different zones based on their metabolic functions [14]. Blood draining from the hepatic arteries and portal veins blends in the hepatic capillaries, also known as sinusoids. The fenestrated structure of the hepatic sinusoids enhances the interaction between the hepatocytes and the circulating blood. The intervening perisinusoidal space, also known as the space of Disse, contains hepatic stellate cells (HSC) and mononuclear cells [15]. In response to chronic injury, HSCs and portal fibroblasts proliferate and transform into myofibroblasts that can contract and secrete ECM [1, 16]. The contracting cells increase the sinusoidal resistance, ultimately leading to portal hypertension [17]. In addition, the resulting ECM-containing scar tissue fills the space of Disse and interrupts fenestrations [18]. Subsequent development of regenerative nodules surrounded with fibrous bands defines cirrhosis.

### Normal anatomy of secondary pulmonary lobule and effect of fibrosis

The functional unit of the lung is the secondary pulmonary lobule—a bronchiole and pulmonary artery branch supply this polyhedral-shaped structure. Although variable in shape, lobules typically have a hexagonal shape in the central part of the lung. This structure is surrounded by connective tissue, also referred to as interlobular septa, which houses pulmonary veins and lymphatics [19]. The secondary pulmonary lobule consists of varying numbers of acini [19, 20]. The acinus is responsible for gas exchange and is located distal to a terminal bronchiole. The Acini are supplied by first-order respiratory bronchiole or bronchioles, the largest airways with alveoli in their walls [19].

The alveolar epithelium is lined with type I and type II alveolar epithelial cells, also known as pneumocytes. AEC1s cover almost 95% of the alveolar epithelium and are mainly responsible for gas exchange, whereas regenerative AEC2s occupy 5% and maintain the integrity of the alveolar epithelium and secrete surfactant. In response to repetitive injury, AEC2s are unable to compensate for the excessive loss of AEC1s [21, 22]. The absence of AEC2s leads to diminished surfactant production and subsequent atelectasis, which, when persistent, leads to pulmonary fibrosis [23]. Moreover, maladaptive repair mechanisms lead to the secretion of profibrotic mediators. These mediators stimulate fibroblast-myofibroblast differentiation and epithelial-mesenchymal transition [24]. The nascent cells are contractile and secrete ECM, which is poorly organized and stiffer, resulting in a positive feedback loop that further stimulates myofibroblast activity [25]. The sequence of events, ultimately leading to UIP with honeycombing, defines pulmonary fibrosis.

### Common features

Profibrotic mediators, mainly transforming growth factor-β (TGF-β) and platelet-derived growth factor (PDGF), are released in response to repetitive damage in both organs, which stimulate myofibroblasts with similar characteristics [26, 27]. Myofibroblasts are the main contributors to the dysregulated ECM in both diseases by expressing a-smooth muscle actin and leading to contraction and stiffness [28, 29]. Type I and type III collagens are predominant in the ECM of both disorders [23, 30], and the stiffer ECM creates a positive feedback loop that further stimulates myofibroblast proliferation and increases their activity [30, 31]. Although this fibrotic response is initially counterbalanced by proteolytic enzymes that degrade the resulting ECM, such as matrix metalloproteinases (MMPs), their capacity is overwhelmed due to chronic injury. Subsequently, myofibroblasts synthesize TIMPs that counteract the effects of MMPs [32, 33].

## Hepatocytes vs. Alveolar epithelial cells

### Hepatocytes

Hepatocytes are the primary cells of the liver, responsible for its metabolic and synthesis functions. The metabolic functions vary along the porto-central axis within the hepatic lobule. From the portal triad to the central vein, gluconeogenesis, β-oxidation, and oxygen concentration decrease gradually, whereas glycolysis, lipogenesis, and triglyceride synthesis increase. Hepatocytes possess cytochrome P enzymes (CYP), which are responsible for the degradation of xenobiotics and other toxins [34, 35]. Hepatocytes are capable of regenerating and repairing the injured liver [34–36]. Repeat damage overwhelms the regenerative capacity of hepatocytes; damaged hepatocytes release reactive oxygen species (ROS) and fibrogenic mediators that trigger inflammation [37]. Subsequently, damaged hepatocytes undergo apoptosis, which activates the HSCs into myofibroblasts and amplifies the fibrogenic activity of myofibroblasts [26, 38]. Accumulation of ECM forms fibrous scars replacing hepatocytes.

### Alveolar epithelial cells

AEC2s possess CYP enzymes, which are responsible for metabolizing inhaled xenobiotics and other toxins [39]. Repeat damage overwhelms the regenerative capacity of AEC2s, and maladaptive repair mechanisms take place [22, 40–42]. Subsequent release of ROS and fibrogenic mediators induce apoptosis of alveolar epithelial cells and the formation of myofibroblasts. Accumulation of ECM forms fibrous scars that destroy normal lung architecture. Consequently, histologic development of UIP and honeycombing defines pulmonary fibrosis.

## Circulation of the liver and lung

### Circulation of liver and portal hypertension

One detrimental consequence of liver cirrhosis is portal hypertension. Portal hypertension develops in response to increased intrahepatic vascular resistance in liver cirrhosis [43, 44]. Normally, liver sinusoidal endothelial cells (LSEC) secrete vasodilator (e.g., nitric oxide) and vasoconstrictor (e.g., endothelin-1, thromboxane A2) substances that regulate sinusoidal resistance. However, liver cirrhosis leads to dysfunctional LSECs, resulting in decreased production of vasodilators and increased production of vasoconstrictors in the liver. Activated HSCs in liver cirrhosis show a diminished response to vasodilators, e.g., nitric oxide (NO). Additionally, increased tortuosity of the liver caused by excessive deposition of ECM and the contraction of myofibroblasts increase the sinusoidal resistance [45]. Unlike intrahepatic vasculature, splanchnic vasculature undergoes vasodilation due to increased NO production, creating a hyperdynamic circulation that increases portal inflow and aggravates portal hypertension [44, 46–48]. VEGF- and PDGF-induced angiogenesis is also considered to contribute to the exacerbation of portal hypertension by enhancing hyperdynamic splanchnic circulation in liver cirrhosis [49].

### Circulation of lung and pulmonary hypertension

One detrimental consequence of pulmonary fibrosis is pulmonary hypertension. The key features of pulmonary hypertension in pulmonary fibrosis are vasoconstriction and vascular remodeling. Production of NO is reduced, while endothelin-1 is increased, leading to pulmonary artery contraction [50]. Unlike systemic circulation, alveolar hypoxia causes vasoconstriction in lung tissue. In addition, hypoxia stimulates vascular remodeling by increasing the proliferation of pulmonary arterial smooth muscle (PASMC) and endothelial cells by increasing the expression of hypoxia-inducible factor 1A [50, 51]. Moreover, the increased deposition of Hyaluronan, a component of ECM in pulmonary fibrosis, and its fragmentation by oxidative stress increases the stiffness of PASMCs in pulmonary fibrosis [50].

## Biliary tree vs. Bronchial tree

### Biliary tree

Primary biliary cholangitis (PBC) and primary sclerosing cholangitis (PSC) are chronic liver diseases that destroy the bile ducts, lead to fibrosis, and eventually cause cirrhosis. Both conditions are classified as cholestatic liver diseases leading to liver injury and fibrosis due to bile stasis. PBC is an autoimmune liver disease that selectively affects the small- to medium-sized intrahepatic bile ducts, particularly the interlobular bile ducts. Non-suppurative cholangitis results from the infiltration of lymphocytes and leads to the loss of bile ducts as the PBC progresses [52]. PSC is characterized by multifocal alternating biliary strictures caused by inflammation and fibrosis of large intrahepatic and extrahepatic bile ducts [53].

### Bronchial tree

The apoptosis and/or dysfunction of AEC1 in pulmonary fibrosis leads to insufficient AEC2 that produce surfactant. Reduced surfactant production by dysfunctional AEC2 increases the surface tension, leading to alveolar collapse. The persistent collapse leads to the coalescence of alveolar walls. Additionally, accompanying deposits of collagen and fibrous tissue harden the collapsed alveoli. This process obliterates the alveoli and is called collapse induration. Collapse induration significantly reduces the surface area of the alveolar epithelium while increasing the alveolar wall thickness [54]. As the disease progresses, imaging of the lungs shows increased airway wall thickness, dilation of the airways, and clustered cystic air spaces called honeycombing. Although not fully elucidated, airway dilation was thought to be due to traction bronchiectasis caused by fibrotic contraction and increased collagen deposition. Bronchiolar proliferation has recently been proposed as the underlying mechanism. The honeycombing structure is considered to result from bronchiolization, i.e., continuous bronchiolar epithelial-like cell proliferation replacing the normal alveolar epithelium [54–56].

## Key promoters of fibrosis

### Liver cirrhosis

The development of liver cirrhosis depends on various promoting factors and signaling pathways in response to repetitive damage in genetically susceptible patients. The mitogenic activity of HSCs is enhanced by several factors, such as PDGF (the predominant mitogen in liver cirrhosis), hepatocyte growth factor (HGF), epidermal growth factor (EGF), fibroblast growth factor (FGF) and vascular endothelial growth factor (VEGF) [30, 57]. Similarly, the hypoxic milieu caused by vascular disorganization enhances the activity of PDGF and vascular endothelial growth factor (VEGF), which promote fibrogenic and angiogenic responses in liver cirrhosis [58–60]. TGF-β is considered the chief driver of fibrogenesis (Table 1). TGF-β also promotes the transition of HSCs to myofibroblasts, induces apoptosis of hepatocytes, increases ECM synthesis, and impedes ECM degradation by downregulating MMPs and other collagenases [57]. In addition, chemokines regulate inflammation by stimulating leukocyte and fibrogenic cell migration. In liver cirrhosis, the expression of CCRs, CCLs, CXCRs, and CXCLs deviates, resulting in a disturbed balance of pro-fibrotic and anti-fibrotic molecules [26, 61, 62]. Subsequently, the tissue damage in the liver stimulates the production of interleukins (ILs). ILs are messenger molecules expressed by a variety of cells, including liver cells. IL-1 activates HSCs, which subsequently drive fibrogenesis in the liver.

**Table 1:** Comparison of fibrotic properties

Effect of Cirrhosis and Fibrosis	Liver	Lung
Main Profibrotic Chemokine	•Transforming Growth Factor-β1	•Transforming Growth Factor-β1
Myofibroblast origin	•Hepatic Stellate Cells •Fibroblast-Myofibroblast Differentiation •Fibrocytes from the Bone Marrow	•Epithelial-Mesenchymal Transition •Fibroblast-Myofibroblast Differentiation •Fibrocytes from the Bone Marrow
Myofibroblast properties	•Contractile •Secretion of extracellular matrix	•Contractile •Secretion of extracellular matrix
ECM composition	Denser, Stiffer, and Dysregulated	Denser, Stiffer, and Dysregulated
Collagen Type	Mainly Type I and III	Mainly Type I and III
Anti-Fibrotic Response	Matrix Metalloproteinases	Matrix Metalloproteinases

### Pulmonary fibrosis

The development of pulmonary fibrosis depends on several promoting factors and signaling pathways in response to repeated damage in genetically susceptible patients. The proliferation of fibroblasts is stimulated by various factors, e.g.: PDGF, TGF-β, fibroblast growth factor (FGF), and endothelial growth factor (EGF) [63, 64]. VEGF-A165a promotes angiogenesis and fibroblast proliferation, whereas VEGF-A165b exerts protective mechanisms in pulmonary fibrosis [63]. Similarly, TGF-β is considered the most important mediator of fibrogenesis and epithelial mesenchymal transition (EMT), which is the transdifferentiation of epithelial cells into fibroblasts/myofibroblasts. In addition, TGF-β induces epithelial cell apoptosis, increases ECM deposition, and impedes ECM degradation by downregulating MMPs and other collagenases [32, 65]. Altered expression of CCRs, CCLs, CXCRs, and CXCLs contributes to the development of pulmonary fibrosis by promoting fibrotic responses and disturbing the balance between angiogenic and angiostatic factors [62, 66]. IL-1α and IL-1β are proinflammatory cytokines, are increased in pulmonary fibrosis, and stimulate fibroblast proliferation.

## Risk factors for cirrhosis and pulmonary fibrosis

### Genetics

**Fig. 1:**
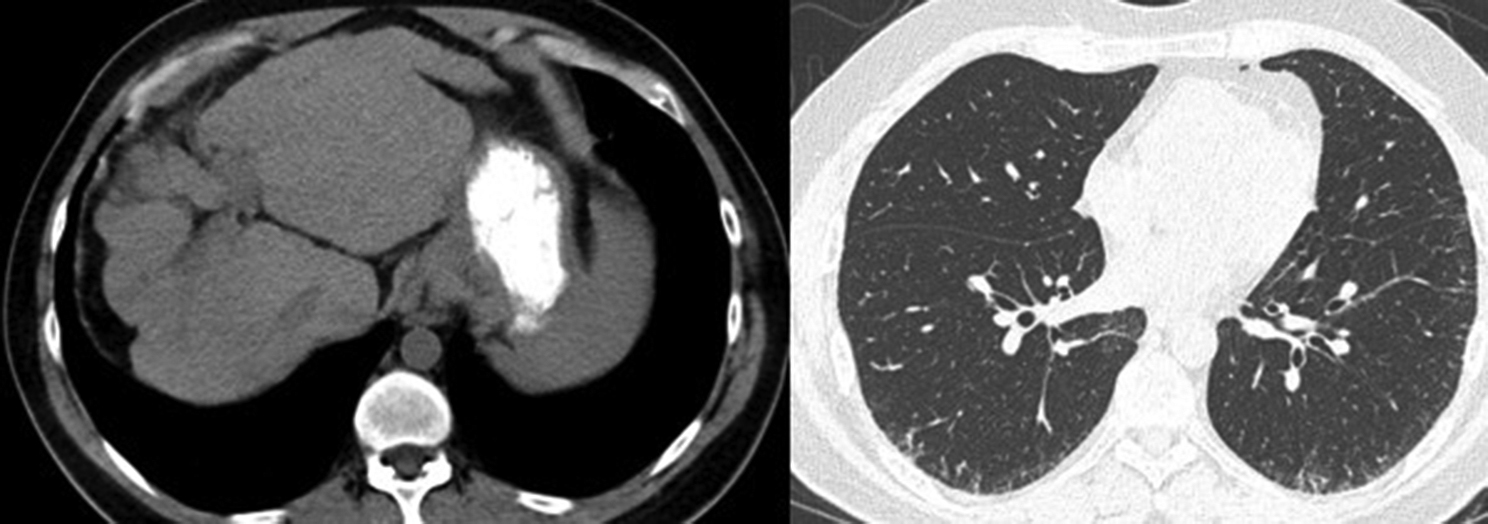
39-year-old male with liver cirrhosis and pulmonary fibrosis suspicious for short telomeres

Numerous genetic studies have shown how mutations contribute to the development of liver cirrhosis and pulmonary fibrosis. Telomerases are known to restore telomere length and halt cell senescence following the replication process. Some studies have shown a correlation between short telomeres and the aforementioned diseases [67–72]. Furthermore, mutations in *TERT* and *TERC* genes, which encode telomerase components, impair telomerase activity and contribute to this shortened structure and subsequent cell senescence and accelerated fibrosis [67, 68, 71–74]. There have been reports of both diseases co-occurring in patients with *TERT* mutations and/or short telomeres [75–77] (Fig. 1).

### Environment

Environmental exposures play an important role in both diseases since they are potentially modifiable. Smoking causes micro-injuries to the lung and activates maladaptive repair mechanisms, causing fibrosis [9, 78–82]. Moreover, smoking-induced hypoxia with secondary polycythemia leads to iron accumulation in hepatocytes and increases the production of inflammatory cytokines (IL-1, IL-6, TNF-a), resulting in necroinflammation and fibrosis of the liver [83, 84]. Smoking also hastens telomere shortening and cellular senescence in a dose-dependent manner, further contributing to fibrosis in both organs [71, 74, 85, 86]. In addition, smoking exacerbates the severity of fibrotic lesions in hepatitis C virus (HCV) patients and accelerates cirrhosis in hepatitis B virus (HBV) patients [87, 88].

Chronic infections lead to persistent damage and fibrosis in both organs. HBV and HCV are the main instigators for the development of cirrhosis [89–91]. Similarly, Epstein-Barr virus (EBV), Human Herpes virus (HHV), Cytomegalovirus (CMV), and HCV have been associated with pulmonary fibrosis. Fibrotic diseases of the liver and lungs are also associated with occupational and agricultural exposures. Some studies have found an association between pulmonary fibrosis and agriculture/farming, livestock, birch dust, and hardwood dust [92, 93]. Cirrhosis, on the other hand, is associated with Aflatoxin exposure and alcohol consumption [94–96].

## Risk of cancer

### Liver cirrhosis and cancer

The vast majority of hepatocellular carcinomas (up to 90%) develop in patients with cirrhosis [97, 98]. HSCs, myofibroblasts, and cancer-associated fibroblasts are thought to contribute to carcinogenesis in liver cirrhosis [99]. In response to hypoxia caused by tumor growth, HSCs secrete angiogenic factors, e.g., VEGF and angiopoietin, promoting tumor vascularization [100–102]. Programmed cell death protein-1 (PD-1) is expressed on the surface of T-cells, B-cells, and natural killer cells and regulates T-cell activity [103, 104]. When bound to the programmed cell death protein-1 ligand (PD-L1), PD-1 acts as an inhibitory molecule. Activated HSCs impair immune surveillance by expressing PD-L1, limiting the immune response against malignant cells [105].

TGF-β plays a bipartite role, exerting tumor-suppressive properties in the early stages of cancer development while acquiring a tumor-promoter status during cancer progression [106]. Continuous TGF-β stimulation causes EMT, leading to resistance to therapy, metastasis, the formation of cancer stem cells, and cancer-associated fibroblasts (CAF) [107, 108]. CAFs, in turn, increase TGF-β gene expression and secrete growth factors (HGF, FGF, EGF) and cytokines (IL-1, IL-6) that stimulate the proliferation of malignant cells [109, 110]. This chronic inflammatory state, caused by the continuous release of cytokines and recruitment of inflammatory cells, results in ROS production by activated cells (i.e., macrophages and HSCs) and damaged hepatocytes [111, 112]. Subsequently, the oxidative stress from ROS induces DNA damage and leads to mutations that promote hepatocellular carcinoma (HCC) [111, 112].

### Pulmonary fibrosis and cancer

In IPF patients, the prevalence of lung cancer varies from 2.7% to 48%, and the incidence increases every year after the diagnosis of IPF [113–116]. PD-1/PD-L1 immune checkpoint axis is induced in IPF, suggesting a possible link between IPF and lung cancer and a mechanism for immune evasion of neoplastic cells in IPF [117–119]. TGF-β is an indispensable mediator of carcinogenesis in IPF. TGF-β suppresses tumorigenesis in the early stages of cancer while later acquiring tumor promoter status [28, 120]. TGF-β also induces the recruitment of myofibroblasts that surround tumor tissue and disrupt the membranes of surrounding tissue, facilitating invasion [121]. TGF-β induces EMT, leading to therapeutic resistance and the formation of CAFs, which are the main components of the tumor microenvironment and promote tumor growth and inflammation through the secretion of various growth factors (FGF, HGF, VEGF), cytokines, and chemokines, e.g., IL-6 and TGF-β [122]. In addition, ROS production by AEC2 enhances the production of profibrotic cytokines and induces oxidative stress, leading to DNA damage and alterations in methylation patterns that promote carcinogenesis [123]. VEGF, which is present in both fibrosis and cancer, is secreted by neoplastic cells to promote angiogenesis and tumor growth [124, 125].

## Similarities

**Fig. 2:**
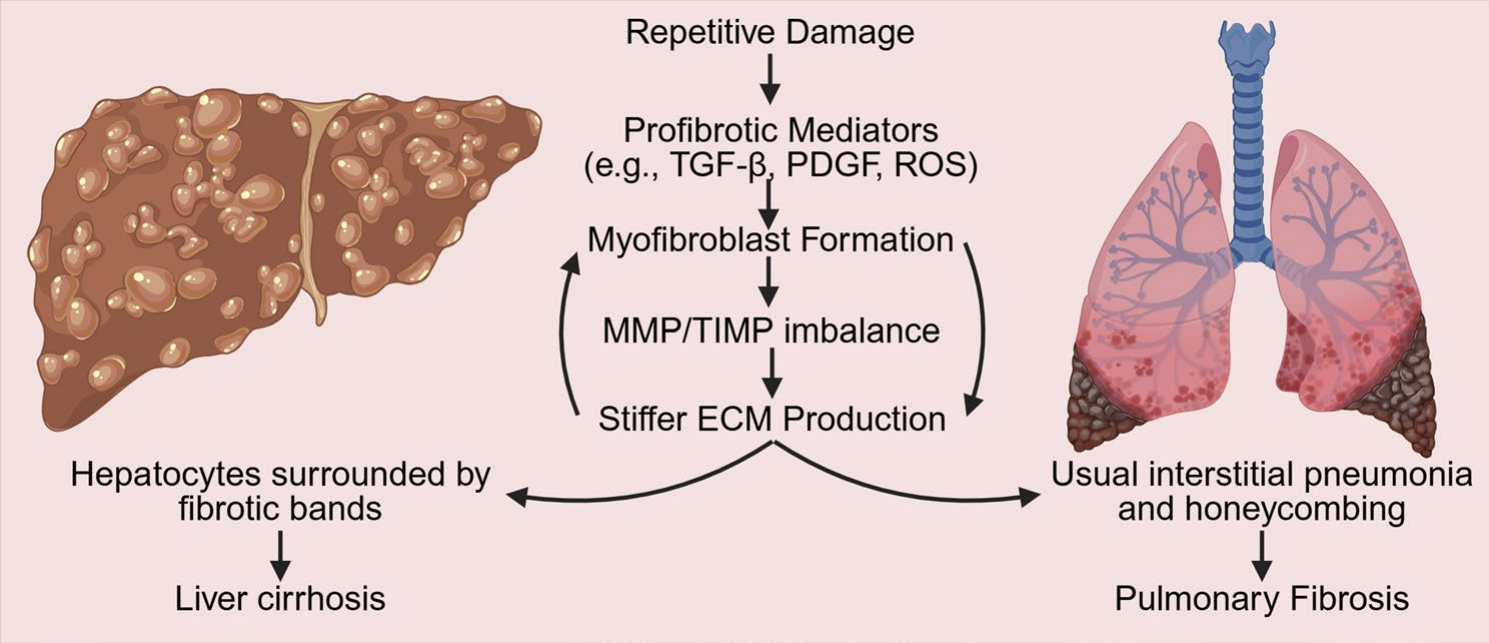
Illustration of the disease development processes in cirrhosis and pulmonary fibrosis. TGF-β: transforming growth factor-β, PDGF: platelet derived growth factor, ROS: Reactive oxygen species, MMP: Matrix metalloproteinases, TIMP: tissue inhibitors of metalloproteinases, ECM: Extracellular matrix

Chronic injury overwhelms the regenerative capacity of regenerative cells (i.e., Hepatocytes, AEC2s) and stimulates the formation of contractile myofibroblasts that secrete poorly organized stiff ECM in both organs (Fig. 2). Simultaneous formation of ROS and secretion of fibrogenic mediators lead to inflammation and apoptosis of hepatocytes and alveolar epithelial cells in the liver and lungs, respectively. Myofibroblasts may arise from fibroblast-myofibroblast differentiation and fibrocytes from bone marrow in both organs. Myofibroblasts also arise from HSCs in the liver and epithelial-mesenchymal transition in the lungs. The ECM formed in both conditions is rich in type I and III collagen and forms fibrous scars that replace and destroy the normal structure of both organs.

TGF-β is the principal inducer of fibrogenesis by inducing apoptosis, myofibroblast formation, and ECM deposition and it prevents ECM degradation by downregulating collagenases.

There is an increased risk of cancer in pulmonary fibrosis and liver cirrhosis. TGF-β stimulates EMT, leading to the formation of CAFs. CAFs secrete various growth factors (e.g., HGF, FGF) and cytokines that promote malignant proliferation and positive feedback TGF-β secretion.

NADPH oxidases (NOXs) mainly generate ROS. In both diseases, the increased production of ROS promotes the development of malignancies. ROS lead to DNA damage and disrupt DNA repair mechanisms, resulting in mutations and, ultimately, HCC. NOX4 expression is upregulated in pre-malignant fibrotic diseases, leading to lung and liver cancer [126].

## Treatments

### Management of liver cirrhosis

Alcohol is one of the leading causes of cirrhosis, and the risk of cirrhosis increases in women with any amount of alcohol consumption and in men with consumption of more than 1 drink per day [127]. Due to anxiety and cravings, patients with alcoholism are prone to relapse. To suppress cravings, baclofen is recommended to maintain abstinence [128, 129]. Non-alcoholic fatty liver disease (NAFLD), particularly its advanced form called nonalcoholic steatohepatitis (NASH), may lead to cirrhosis and can be managed by weight loss. Weight loss of ≥ 5% resulted in the resolution of NASH in 58% of patients, and this ratio increased to 90% in patients with weight loss of ≥ 10%. In the latter group, fibrosis regression occurred in 45% of the patients. Additionally, chronic HBV and HCV infections are important causes of liver cirrhosis. In a systematic review published by Alberts et al., it was found that of all cirrhotic patients, 42% had HBV infection and 21% had HCV infection [130]. The use of antiviral agents against HBV and HCV leads to a reduction in inflammation and fibrosis [131–133]. Nonselective beta-blockers are used as primary and secondary prophylaxis for a serious complication associated with portal hypertension, namely variceal hemorrhage, which is a leading cause of death in cirrhosis [129, 134–136]. In addition, transjugular intrahepatic portosystemic shunt (TIPS) placement can be performed to lower portal pressure in patients at high risk of bleeding, as secondary prophylaxis after acute hemorrhage, or as salvage therapy for refractory variceal bleeding [137].

To date, there is no FDA-approved antifibrotic therapy for liver cirrhosis. Some trials investigate the antifibrotic effect via various mechanisms, such as inhibition of hepatocyte apoptosis, reduction of oxidative stress, inhibition of HSC activation, reduction of fibrotic scar development and contraction, and immune modulation [26] (Table 2). Although pirfenidone is not approved for cirrhosis, some studies are investigating the anti-fibrotic efficacy of the drug in the liver [147, 148]. There is also a pirfenidone analog, Hydronidone, which is under phase 3 trial [149] and has shown promising results in a phase 2 clinical trial with achieving significantly higher proportion of fibrosis regression at 52 weeks. [145, 146] Liver transplantation remains the only curative option for end-stage liver disease and cirrhosis. The five-year survival rate for recipients of liver transplants from deceased donors is approximately 75% [150].

**Table 2:** Results of recent clinical trials for liver cirrhosis

Agent	Study Design	Result of the primary outcome
Emricasan (Pan-caspase inhibitor)	IDN-6556, NCT02138253 [138] Phase 2: Post-Transplant Chronic HCV infection Primary Outcome: Number of participants with at least a one-stage reduction from baseline in Ishak Fibrosis Stage within 24 months	Risk Difference: 2.9 [−20.5, 26.4] (*p* = 0.73)
Setanaxib (Dual NOX1/4 inhibitor)	GKT13783, NCT03226067 [139, 140] Phase 2: Assessing the safety and efficacy of Setanaxib in patients with PBC who are taking a stable dose of ursodeoxycholic acid treatment, and have persistently high levels of ALP Primary Outcome: The percent change in serum GGT from baseline to week 24	The mean percent change (±SD) was −4.9% (59.6) in once daily arm, −19.0% (28.9) in twice daily arm, and −8.4% (21.5) in the placebo arm.
PRI-724 (CBP/β-catenin small molecule inhibitor	PRI-724, NCT03620474 [141, 142] Phase 1/2a: investigating the safety and anti-fibrotic effects of PRI-724 in patients with HBV- and HCV-induced cirrhosis Primary Outcome of Phase 1: safety, tolerability, and dose-limiting toxicities of multiple escalating doses of PRI-724 when administered via IV infusion Primary Outcome of Phase 2a: efficacy of liver cirrhosis treatment (assessed by examining changes related to baseline in liver tissue fibrosis according to liver biopsy)	•Intravenous administration of 280 mg/m^2^/4 h PRI-724 over 12 weeks was well tolerated. •The most common adverse events were diarrhea and nausea. •Ordinal scoring or measurement of collagen proportionate area at 12 weeks did not reveal a significant reduction in hepatic fibrosis. •Significant improvements in liver stiffness, Model for End-stage Liver Disease score, and serum albumin levels were observed.
BMS-986263	BMS-986263, NCT03420768 [143, 144] Phase 2: In adults with advanced hepatic fibrosis due to HCV who had achieved sustained virologic response Primary Outcome: Proportion of patients with ≥ 1 stage improvement in Liver Fibrosis (METAVIR Score) at 12 weeks’ biopsy	The METAVIR improvement ratio was 16.7% in the 45 mg once-weekly arm, 21.4% in the 90 mg once-weekly arm, and 13.3% in the placebo once-weekly arm.
Hydronidone (Pirfenidone Analog)	F351, NCT02499562 [145, 146] Phase 2: Patients with chronic HBV related liver fibrosis were randomized to receive hydronidone or placebo capsules once daily for 52 weeks along with entecavir Primary Outcome: Proportion of patients achieving an improvement of ≥ 1 point in the Ishak fibrosis stage from baseline after 52 weeks of treatment.	270 mg Hydronidone achieved a significantly higher proportion of patients achieving ≥1-stage fibrosis regression at Week 52 compared with placebo (54.8% vs. 25.6%, *p* = 0.006).

### Management of pulmonary fibrosis

The risk of developing IPF was significantly associated with a history of ever smoking (odds ratio [OR] = 1.6; 95% CI = 1.1–2.4) [151]. IPF patients who had never smoked lived longer than current and former smokers with IPF (hazard ratio [HR] = 0.63; 95% CI = 0.45–0.90; *p* < 0.01) [152]. Smoking cessation is, therefore, essential at any stage of the disease.

In recent years, several publications have investigated the relationship between IPF prognosis and physical activity levels. Vainshelboim et al. [153] reported that IPF patients who sat > 5 h/day had experienced a higher risk of hospitalization and mortality than patients who reported sitting < 5 h/day. Patients who walked more than 100 minutes per week had a lower risk of hospitalization and mortality. Although the significance vanished after adjustment for forced vital capacity (FVC) and diffusion capacity of the lungs for carbon monoxide (DLCO), this change was attributed to the small sample size and a low number of deaths for the multivariate model.

Some infections, e.g., EBV, HHVs, CMV, and Streptococcus pneumoniae, have been identified in IPF patients. Recently, Sheng et al. [154] published a meta-analysis showing that chronic infections with these viruses (CMV, EBV, and HHVs) are associated with a significantly increased risk of developing IPF (odds ratio [OR] including all viruses in the study: 3.48; 95% CI: 1.61–7.52; *p* = 0.001). In EBV IgG-positive patients, 2-week treatment with ganciclovir has attenuated the progression of IPF [155]. When treated with amoxicillin or clarithromycin, Streptococcus pneumoniae-infected mice had lower hydroxyproline levels, which is a measure of lung collagen content [156].

Concomitant pulmonary hypertension correlates with mortality and is therefore associated with prognosis in IPF patients. Lettieri et al. [157] reported that the one-year mortality rate was higher in patients with IPF-pulmonary hypertension than with IPF alone (28% and 5.5%, respectively; *p* = 0.002). Endothelin receptor antagonists, prostanoids, phosphodiesterase type 5 inhibitors, and soluble guanylate cyclase inhibitors are used to lower pulmonary arterial pressure. However, a recent meta-analysis failed to demonstrate a significant difference in all-cause mortality between the pulmonary hypertension-specific agent receiving and control groups in IPF patients (HR = 0.99; 95% CI = 0.92–1.06; *p* = 0.71) [158].

The 2011 ATS/ERS/JRS/ALAT statement [159] rejects the use of corticosteroid monotherapy and corticosteroids in combination with immunomodulators in IPF but recommends the use of corticosteroids in acute exacerbation of IPF. Given the increased incidence of venous thromboembolic events in IPF patients (relative risk [RR] = 2.11; 95% CI = 1.28–3.48) [160], the use of anticoagulants is under investigation. The 2015 ATS/ERS/JRS/ALAT statement [161] strongly rejects anticoagulation with warfarin unless there is an alternative indication. King et al. [162] reported that warfarin use was associated with a reduction in transplant-free survival (HR = 2.101; 95% CI = 1.025 - 4.307) in the adjusted analysis as opposed to direct oral anticoagulants (DOACs) (HR = 1.51; 95% CI = 0.71–3.22). Warfarin was also associated with an increased risk of death or transplantation in patients with IPF (HR = 2.57; 95% CI = 1.10–6.02) in the adjusted analysis, whereas direct oral anticoagulants were not (HR = 1.37; 95% CI = 0.50–3.74).

Pirfenidone and nintedanib are the approved antifibrotic agents for IPF. Pirfenidone inhibits TGF-β, collagen synthesis, and fibroblast proliferation and has anti-inflammatory and antioxidant properties [163–166]. Nintedanib inhibits several tyrosine kinase receptors, including the PDGF receptor, FGF receptor, and VEGF receptor. The 2015 ATS/ERS/JRS/ALAT statement [161] recommends pirfenidone and nintedanib in patients with IPF. In IPF patients treated with either pirfenidone or nintedanib, there was a reduction in FVC decline and all-cause mortality [167, 168].

In patients with end-stage IPF, lung transplantation is a life-prolonging procedure. The transplantation can be either unilateral or bilateral. A recent meta-analysis found no significant difference in overall survival between unilateral and bilateral transplantation (HR = 1.08; 95% CI = 0.91–1.29; *p* = 0.391) [169]. However, bilateral transplants were associated with better postoperative pulmonary functions in forced expiratory volume in one second (FEV1)%, FVC %, and DLCO %. An overview of recent clinical trials is provided in Table 3.

**Table 3:** Results of recent clinical trials for pulmonary fibrosis

Agent	Study Design	Results of the primary outcome
Pamrevlumab (Monoclonal Antibody against CTGF)	FG-3019, NCT03955146 [170, 171], NCT04419558 [172] Phase 3: Evaluating the efficacy and safety of pamrevlumab in patients with IPF who are not being treated with approved IPF therapies (pirfenidone, nintedanib) Primary Outcome: Change from baseline in FVC at week 48	The Phase 3 studies were terminated since the study did not meet its primary endpoint. NCT03955146: No statistically significant between the groups (Pamrevlumab vs. Placebo) for the absolute change in FVC from baseline to 48 weeks [170]
Pentraxin (Recombinant Human Pentraxin-2)	PRM-151, NCT02550873 [173, 174] Phase 2: Evaluating the efficacy and safety of PRM-151 Primary Outcome: Change from baseline in FVC (%predicted) at 28-week	The mean change in FVC percentage of predicted value from baseline to 28 weeks [174]: 2.3 (90% CI: 1.1–3.5, *p* = 0.001) [174] Change in PRM-151 10 mg/kg group: −2.5 (−3.2 to −1.8) Change in the Placebo group: −4.8 (−5.7 to −3.8)
	NCT04552899 [175, 176], NCT04594707 [177] Phase 3: Evaluating the long-term safety, efficacy, and pharmacokinetics of pentraxin Primary Outcomes: Change from baseline in FVC at 52-week and observation of adverse events	NCT04552899: Absolute change in FVC (mL) in Zinpentraxin Alfa and Placebo groups was not significantly different [176] (−235.72 [−283.07 to −188.4] vs. −214.89 [−262.44 to −167.3], *p* = 0.54)
Setanaxib (Dual NOX1/4 inhibitor)	GKT13783, NCT03865927 [178] Phase 2: Evaluating the efficacy of the drug and monitoring the safety Primary Outcome: changes in concentrations of a circulating surrogate biomarker for oxidative stress measured by mass spectroscopy	Results Pending
Ziritaxestat (Autotaxin inhibitor)	GLPG1690, NCT03733444 [179, 180], NCT03711162 [180, 181] Primary outcome of Phase 3: Annual rate of decline in FVC up to 52 weeks (Both trials were terminated due to improper benefit-risk profile)	The results represent the least squares mean (CI) annual rate of FVC decline at 52 weeks. NCT03733444 [179, 180]: 600 mg: −173.8 (−209.2 to −138.4) 200 mg: −174.9 (−209.5 to −138.4) Placebo: −176.6 (−211.4 to −141.8) 600 mg vs. Placebo Mean difference: 2.8 (95% CI = −46.9 to 52.4) 200 mg vs. Placebo Mean difference: 1.7 (95% CI = −47.4 to 50.8) NCT03711162 [180, 181]: 600 mg: −124.6 (−178 to −71.2) 200 mg: −173.9 (−225.7 to −122.2) Placebo: −147.3 (−199.8 to −94.7) 600 mg vs. Placebo Mean Difference: 22.7 (95% CI = −52.3 to 97.6) 200 mg vs. Placebo Mean Difference: −26.7 (95% CI = −100.5 to 47.1)
Nerandomilast (Phosphodiesterase 4B inhibitor)	NCT05321069, FIBRONEER-IPF Trial [182, 183] Primary Outcome: Absolute change from baseline in forced vital capacity at 52 weeks	The results represent the least squares mean [CI] rate of FVC decline (ml) at 52 weeks. NCT05321069: 18 mg Nerandomilast: −114.7 [−141.8 to −87.5] 9 mg Nerandomilast: −138.6 [−210.9 to −156.1] Placebo: −183.5 [−210.9 to −156.1] 18 mg vs. Placebo mean difference: 68.8 ml [30.3–107.4, *p* < 0.001] 9 mg vs. Placebo Mean difference: 44.9 [6.4–83.3, *p* = 0.02]

## Future directions and emerging therapies

The similarities we outlined across the two disease processes can serve as a guidance for translational studies to focus on identifying shared biomarkers by integrating multi-omics analyses. Furthermore, the efficacy of antifibrotic agents developed for one fibrotic disease could be systematically evaluated in pre-clinical models of the other, given the shared cellular and molecular mechanisms driving ECM remodeling. For instance, agents approved for idiopathic pulmonary fibrosis, such as nintedanib and pirfenidone, have shown partial efficacy in experimental liver fibrosis models.[184, 185]

Emricasan, a pan-caspase inhibitor, was testedin a phase 2 trial involving patients with post-transplant chronic HCV infection, and failed to demonstrate significant histologic improvement (Table 2) [138]. Setanaxib, a dual NOX1/4 inhibitor, targets reactive oxygen species generation and oxidative stress–mediated fibrogenesis. In a phase 2 study of patients with primary biliary cholangitis receiving ursodeoxycholic acid, twice-daily dosing achieved a greater reduction in serum glutamyl transferase (GGT) compared with placebo. [139, 140] PRI-724, a small-molecule CBP/β-catenin inhibitor, modulates Wnt/β-catenin signaling to limit hepatic stellate cell activation. In a phase 1/2a trial including patients with HBV- or HCV-induced cirrhosis, intravenous administration of PRI-724 for 12 weeks was well tolerated and led to improvements in liver stiffness, Model for End-stage Liver Disease (MELD) score, and serum albumin levels, although no significant reduction in fibrosis stage was observed. [141, 142] BMS-986263, a lipid nanoparticle delivering small-interfering RNA targeting heat-shock protein 47 (HSP47), aims to suppress collagen synthesis in hepatic stellate cells. In a phase 2 trial of patients with advanced hepatic fibrosis secondary to HCV infection who had achieved viral eradication, 16–21% of treated participants exhibited ≥1-stage improvement in METAVIR fibrosis score compared with 13% in the placebo arm. [143, 144] Hydronidone (F351), a novel analog of pirfenidone, exerts antifibrotic effects through inhibition of TGF-β signaling and collagen deposition. In a phase 2 study of patients with chronic HBV-related fibrosis receiving concomitant entecavir, Hydronidone achieved≥1-stage Ishak fibrosis regression in 54.8% of patients compared with 25.6% in the placebo group (*p* = 0.006). [145, 146]

Pamrevlumab, a monoclonal antibody against connective tissue growth factor (CTGF), inhibits extracellular matrix (ECM) deposition and fibroblast activation (Table 3). However, phase 3 clinical trials did not meet their primary endpoint of forced vital capacity (FVC) preservation, and further development has been discontinued. [170–172] Pentraxin-2 (PRM-151), a recombinant human pentraxin that modulates macrophage polarization and fibrosis resolution, demonstrated significant preservation of lung function in phase 2 studies (+2.3% predicted FVC, *p* = 0.001), although subsequent phase 3 trials failed to replicate these results. [173–177] Setanaxib, which has been tested for cirrhosis, is still under investigation for pulmonary fibrosis. [178] Ziritaxestat, an autotaxin inhibitor that disrupts lysophosphatidic acid signaling, was evaluated in two large phase 3 trials that were ultimately terminated due to an unfavorable benefit–risk profile, despite showing comparable FVC trends to placebo. [179, 180] Nerandomilast achieved a statistically significant attenuation of FVC decline compared with placebo in the phase 3 FIBRONEER-IPF trial (mean difference +68.8 mL, *p* < 0.001). [182, 183]

## Conclusions

Cirrhosis and pulmonary fibrosis share molecular and cellular pathways, leading to fibrosis, vascular remodeling and malignant transformation. Recognizing these shared pathways not only refines our understanding of organ-specific fibrotic processes but also highlights potential common therapeutic targets. Insights explored from one disease can inform the pathogenesis and treatment strategies of the other, which can facilitate the identification of cross-organ biomarkers and shared antifibrotic pathways. Moreover, the similarities in the fibrogenic cascades create opportunities to evaluate existing antifibrotic agents. Such cross-disease investigations could accelerate the development of targeted therapies applicable across multiple fibrotic disorders.

## Data Availability

Not applicable

## Abbreviations

IPF: Idiopathic Pulmonary Fibrosis
ECM: Extracellular Matrix
MMP: Matrix Metalloproteinase
AEC: Alveolar Epithelial Cells
UIP: Usual Interstitial Pneumonia
HBV: Hepatitis B virus
HCV: Hepatitis C virus
EBV: Epstein-Barr Virus
CMV: Cytomegalovirus
HHV: Human Herpes Virus
HSC: Hepatic Stellate Cells
TGF-β: Transforming Growth Factor-β
PDGF: Platelet-derived Growth Factor
TIMP: Tissue Inhibitors of Metalloproteinase
CYP: Cytochrome P enzymes
ROS: Reactive Oxygen Species
VEGF: Vascular Endothelial Growth Factor
FGF: Fibroblast Growth Factor
EGF: Endothelial Growth Factor
HGF: Hepatocyte Growth Factor
EMT: Epithelial Mesenchymal Transition
LSEC: Liver Sinusoidal Endothelial Cells
NO: Nitric Oxide
PASMC: Pulmonary Arterial Smooth Muscle Cell
PBC: Primary Biliary Cholangitis
PSC: Primary Sclerosing Cholangitis
PD-L: Programmed Cell Death Protein – Ligand
HCC: Hepatocellular Carcinoma
CAF: Cancer Associated Fibroblast
NAFLD: Non-alcoholic Fatty Liver Disease
NASH: Nonalcoholic Steatohepatitis
TIPS: Transjugular Intrahepatic Portosystemic Shunt
NSAID: Non-steroidal Anti-inflammatory Drugs
FVC: Forced Vital Capacity
DLCO: Diffusion capacity of the lungs for carbon monoxide
OR: Odds ratio
HR: Hazard Ratio
DOAC: Direct Oral Anticoagulants
CI: Confidence Interval
FEV1: Forced Expiratory Volume in one second
FT: FibroTest
FRP: Fibrosis Regression Profile
HAI: Histological Activity Index
HRCT: High Resolution Computed Tomography
UTE-MRI: Ultrashort Echo time MRI
